# Lung metastases from intraductal papillary neoplasm of the bile duct: a case report

**DOI:** 10.1186/s12957-020-02054-9

**Published:** 2020-10-23

**Authors:** Rika Fujino, Yoshihito Masuoka, Taro Mashiko, Akira Nakano, Kenichi Hirabayashi, Toshio Nakagohri

**Affiliations:** 1 Department of Surgery, Tokai University School of Medicine, Shimokasuya 143, Isehara, Kanagawa 2591193 Japan; 2 Department of Pathology, Tokai University School of Medicine, Shimokasuya 143, Isehara, Kanagawa 2591193 Japan

**Keywords:** Intraductal papillary neoplasm of the bile duct, IPNB, Recurrence, Re-resection, Lung metastasis, Surgery

## Abstract

**Background:**

Intraductal papillary neoplasm of the bile duct (IPNB) is considered a pre-cancerous biliary lesion and/or an early cancer lesion, although its classification remains unclear. The 2019 revised edition of the World Health Organization Classification of Tumors of the Digestive System proposed type 1 and type 2 as new classification categories, and meta-analyses and/or multi-center cohort studies are beginning to be reported. However, treatment for IPNB recurrence and metastasis remains unclear.

**Case presentation:**

A 60-year-old man who was referred to our hospital after a suspected liver tumor was diagnosed using abdominal ultrasonography. Imaging findings revealed an irregularly shaped tumor in segment 5 (S5) of the liver (size 20 mm). The S5 lesion was suspected as IPNB, and segmentectomy was performed. The pathological findings revealed invasive carcinoma derived from IPNB, and immunohistochemistry revealed positive expression of MUC1, MUC5AC, and MUC6, but negative expression of CDX2 and MUC2. At 9 months after the surgery, computed tomography revealed a tumor in the right bile duct, which was diagnosed as liver recurrence of IPNB, and right hepatectomy was performed. The histopathological findings were the same as for the first resected specimen (i.e., IPNB). At 45 months after the second surgery, computed tomography revealed nodules in both lungs, which were diagnosed as lung metastases from IPNB and resected in two separate procedures. The pathological findings were metastatic carcinoma from IPNB for both lung lesions. The patient is currently alive and undergoing adjuvant chemotherapy (S-1), which was initiated 64 months after the first resection and 12 months after resection of the lung metastases.

**Conclusion:**

We encountered a rare case of lung metastases from IPNB, which were diagnosed immunohistologically. Because IPNB is generally a slow-growing tumor, resection may be feasible for IPNB recurrence and/or metastasis, which may be detected during long-term follow-up. Thus, even if resection is performed for primary IPNB, additional surgical treatment may be feasible in this setting.

## Background

Intraductal papillary neoplasm of the bile duct (IPNB) is a disease concept that was introduced by Chen et al. to describe mammilliform tumors growing in the intrahepatic and/or extrahepatic bile duct [1]. This entity was described in 2010 as a pre-cancer biliary lesion and/or early cancer lesion in the revised World Health Organization (WHO) Classification of Tumors of the Digestive System. Although Gordon-Weeks et al. published a systematic review and meta-analysis in 2016 [2], the classification of this rare lesion remains unclear, which is associated with most studies involving small retrospective reviews. The 2019 revision of the WHO Classification of Tumors of the Digestive System [3] defined IPNB as an intraductal papillary neoplasm of the liver and bile ducts, which is grossly visible, premalignant, and exhibits intraductal papillary or villous growth of biliary-type epithelium. If an invasive carcinoma component is present, the lesion is designated as intraductal papillary neoplasm with associated invasive carcinoma. IPNB is classified as type 1 (intrahepatic) or type 2 (extrahepatic), which was suggested by a Japanese–Korean study group [4] and adopted by the WHO in 2019 [3].

Although IPNB is a rare tumor, it is thought to have regional variations in its incidence. For example, in eastern Asia, IPNB accounts for 10–38% of bile duct tumors, and the intestinal/gastric types are the most common [3]. There is a minimal risk of recurrence and death in cases involving low-grade dysplasia, although cholangiocarcinoma derived from IPNB is associated with a 5-year recurrence rate of 47.0% and a 5-year overall survival rate of 68.8% [3]. You et al. reported 74 cases with invasive IPNB [5] and noted that local recurrence was the most common first recurrence site, with distal metastasis potentially involving the liver, peritoneum, and retroperitoneal lymph nodes. However, we are only aware of two previous cases involving lung metastasis from IPNB, which were reported by Höhn et al. in a single-center survey [6] and another case report [7]. Thus, because it is unclear what treatment is appropriate for IPNB recurrence and/or metastasis, we report what we believe is the third case of lung metastasis from IPNB.

## Case presentation

**Fig. 1:**
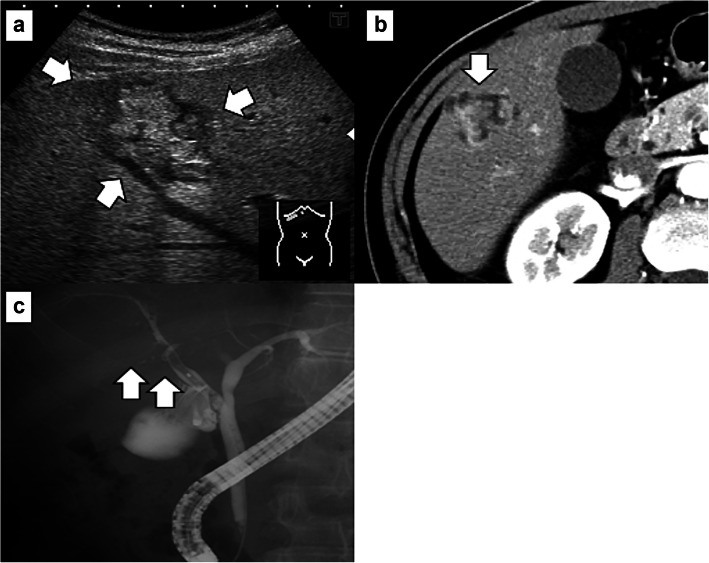
Preoperative imaging findings. **a** Abdominal ultrasonography revealed a hyperechoic tumor (core size of 20 mm) in the intrahepatic area and peripheral to the hypoechoic area (white arrow). The hyperechoic area is even more intense at the posterior aspect of the tumor. **b** Dynamic computed tomography revealed an unevenly shaped and enhanced tumor (white arrow) in S5 of the liver during the arterial phase. **c** Endoscopic retrograde cholangiography revealed a filling defect (white arrow) from the origin of the right area to the periphery and severe dilation of the S5 branch of the bile duct

**Fig. 2:**
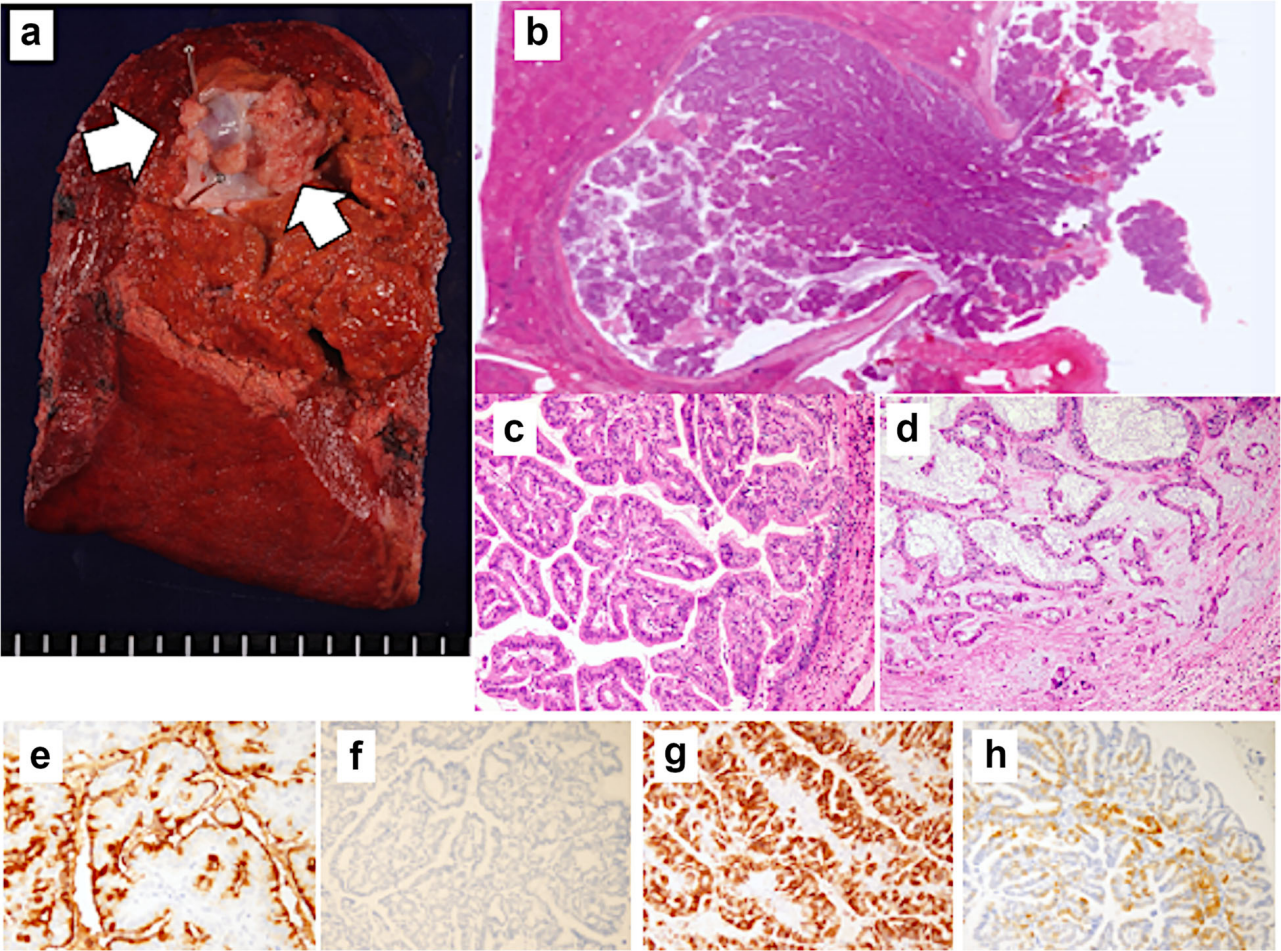
Macroscopic and pathological findings from the first resection. **a** The papillary and nodular tumor in the dilatation of intrahepatic bile duct (white arrow). **b** Complex papillary or tubular proliferation of atypical columnar or cuboidal cells with focal fine vascular stroma was observed in the dilated intrahepatic bile ducts. **c** Most of the tumor was composed of high-grade atypical cells that had enlarged nuclei, whereas low-grade dysplasia components were focally observed. **d** Tumor focally invaded into the stroma of portal area with mucinous trabecular or tubular fashion, although there was no invasion to the liver parenchyma. Immunohistochemistry findings are shown for **e** MUC1, **f** MUC2, **g** MUC5AC, and **h** MUC6

A 60-year-old man was referred to our hospital after a suspected liver tumor was identified using abdominal ultrasonography. The patient did not exhibit any symptoms at the referral, although he was receiving medication to manage hypertension and hyperlipemia. Laboratory test results revealed normal findings for carcinoembryonic antigen (CEA, 10.7 ng/mL) level, cancer antigen 19-9 (CA19-9, 33.1 U/mL) level, and protein induced by vitamin K absence or antagonist-II (26 mAU/mL) level. Abdominal ultrasonography showed an irregularly shaped tumor in segment 5 (S5) of the liver, with a hyperechoic core (size 20 mm) in the intrahepatic area and peripheral to the hypoechoic area. The hyperechoic area was even more intense at the posterior aspect of the tumor (Fig. 1a). Abdominal dynamic computed tomography (CT) revealed an irregularly shaped and enhanced tumor in S5 of the liver during the arterial phase (Fig. 1b). Endoscopic retrograde cholangiography (ERC) revealed a filling defect from the origin of the right area to the periphery and severe dilation of the S5 branch of the bile duct (Fig. 1c). ERC showed a mucus discharge from the ampulla of Vater; however, we were not able to confirm adenocarcinoma via brushing cytology from the bile duct, doubting the presence of a mucus-producing tumor (especially IPNB). Therefore, we decided on surgery, performing segmentectomy for the S5 lesion. We decided to perform segmentectomy because the result of the rapid diagnosis made based on the bile duct stump during surgery was negative. The resected liver showed the papillary and nodular tumor in the dilatation of the intrahepatic bile duct (Fig. 2a). Microscopically, complex papillary or tubular proliferation of atypical columnar or cuboidal cells with focal fine vascular stroma was observed in the dilated intrahepatic bile ducts (Fig. 2b). Most of the tumor was composed of high-grade atypical cells with enlarged nuclei, whereas low-grade dysplasia components were focally observed (Fig. 2c). The tumor focally invaded into the stroma of the portal area in mucinous trabecular or tubular fashion but without invasion to the liver parenchyma (Fig. 2d). Immunohistochemically, MUC1 and MUC5AC were diffusely positive, and MUC6 was focally positive; however, MUC2 was negative (Fig. 2e–h). From these histological features, we diagnosed as IPNB associated with invasion (pT1aN0M0 stage IA, the 8th edition of UICC). From the tumor, mainly composed of high-grade atypical cells with complex papillary or tubular structure, and immunohistochemical pattern of MUC expressions (MUC1+/MUC2-/MUC5AC+/MUC6+), the subtype was considered as pancreatobiliary. In addition, because the tumor was located in the intrahepatic bile ducts, and histological features were similar to pancreatic intraductal papillary mucinous neoplasm, we considered it IPNB type 1. There was no lymph node or distant metastasis. Adjuvant chemotherapy was not provided because we observed no histopathological evidence of residual tumor.

**Fig. 3:**
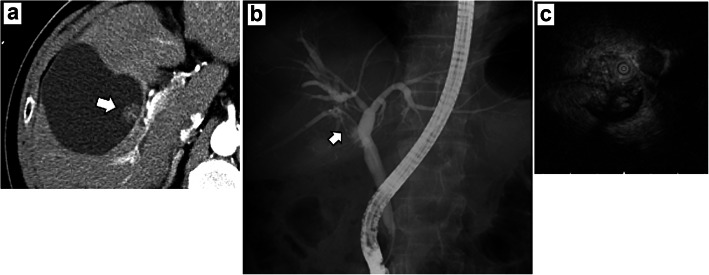
Imaging findings from the liver recurrence. **a** Dynamic computed tomography revealed an enhanced tumor within a cystic substance during the arterial phase (white arrow). **b** Endoscopic retrograde cholangiography revealed a filling defect (white arrow). **c** Intraductal ultrasonography showed a papillary tumor in the dilatation of right hepatic duct

**Fig. 4:**
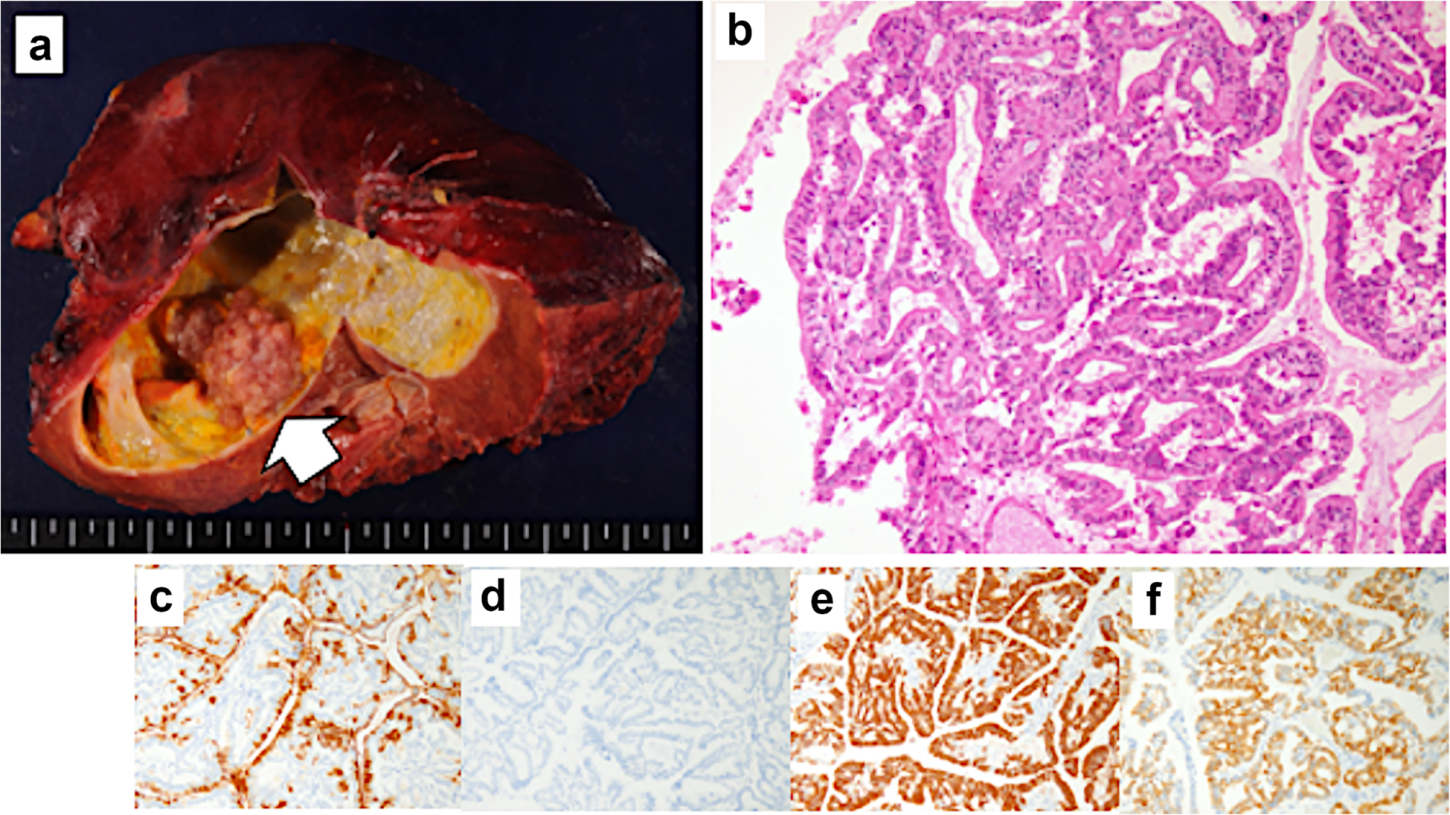
Macroscopic and pathological findings from the recurrence. **a** Macroscopic findings from the recurrence revealed a papillary tumor in the cystic tumor (white arrow). **b** Hematoxylin and eosin staining revealed papillary growth at the epithelium and abundant mucus production. Immunohistochemistry findings are shown for **c** MUC1, **d** MUC2, **e** MUC5AC, and **f** MUC6

The patient remained in good condition until CT revealed recurrence in the right hepatic duct at 9 months after the surgery (Fig. 3a). ERC revealed a filling defect in the origin of the right bile duct, and intraductal ultrasonography (IDUS) revealed a papillary tumor (Fig. 3b, c). We diagnosed the patient with intrahepatic recurrence of IPNB and performed right hepatectomy. Macroscopically, the papillary and nodular tumor projected into the dilated intrahepatic bile duct (Fig. 4a). Microscopic examination showed papillary or tubular proliferation of atypical columnar or cuboidal cells in the dilated intrahepatic bile duct (Fig. 4b). Immunohistochemical patterns showed MUC1, MUC5AC, and MUC6 positive and MUC2 negative (Fig. 4c–f). These findings were similar to the first resection. From these findings, we diagnosed IPNB recurrence. Adjuvant chemotherapy was not re-administered on the basis of our judgment that curative resection has been achieved. The patient underwent follow-up CT examinations every 3 months for the first 2 years after the second surgery, and then underwent follow-ups every 6 months using CT and testing for serum tumor markers (CEA and CA19-9).

**Fig. 5:**
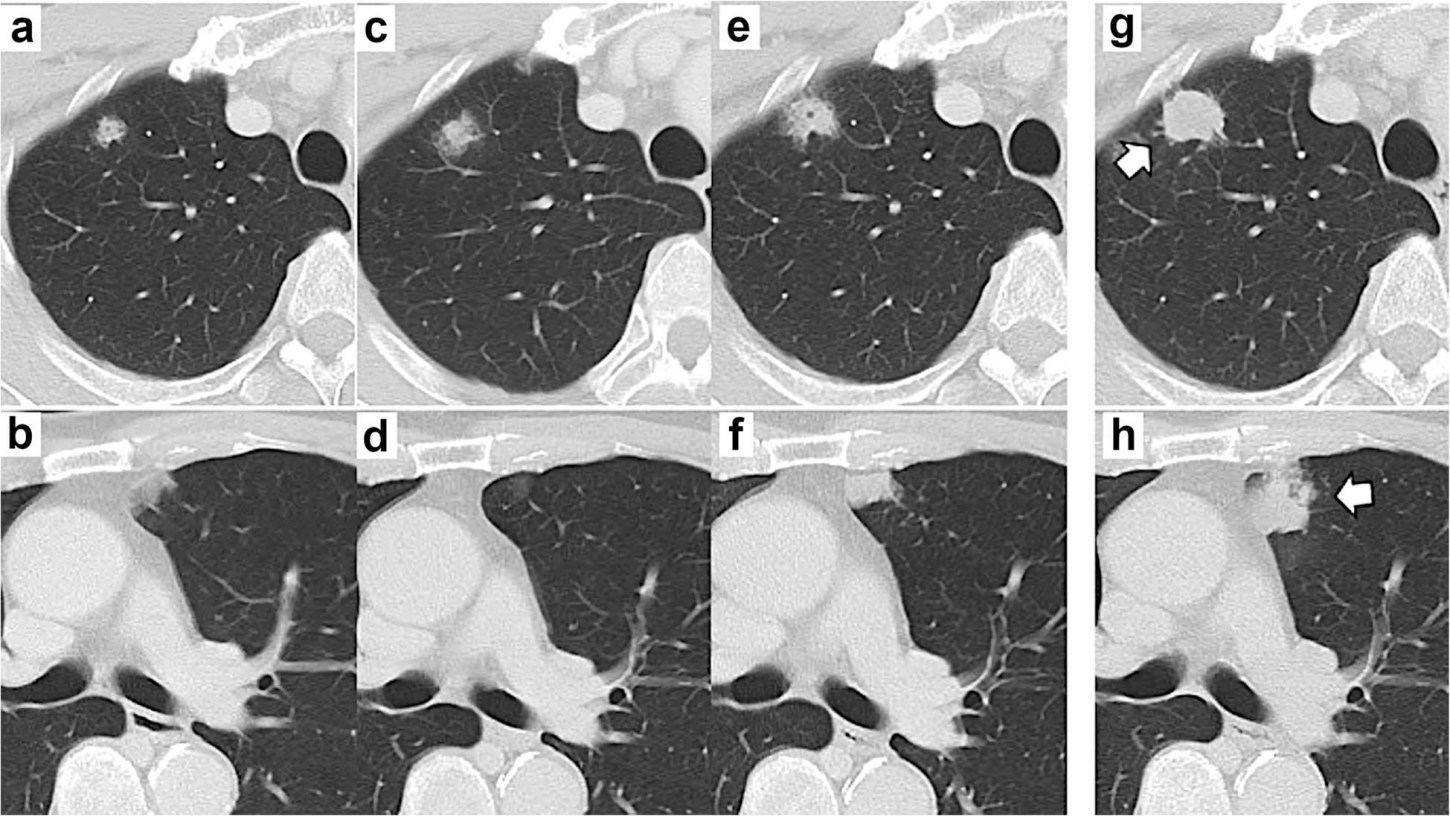
Imaging findings for both of the lung metastases. Computed tomography showed a 5-mm-sized frosted glass shadow in the right lung apex (**a**), and a 5-mm-sized node from the left superior mediastinum to the ventral pleura in the left lung (**b**) 42 months after first resection. Forty-six months after first resection, the right lesion changed to a nodule and expanded to 10 mm (**c**), the left lesion disappeared (**d**). Both the right (**e**) and left lesions (**f**) indicated growth again 50 months after first resection. The size of both tumors, the right (**g**: white arrow) and left lesion (**h**: white arrow) had increased to 20 mm right before resection 54 months after first resection

**Fig. 6:**
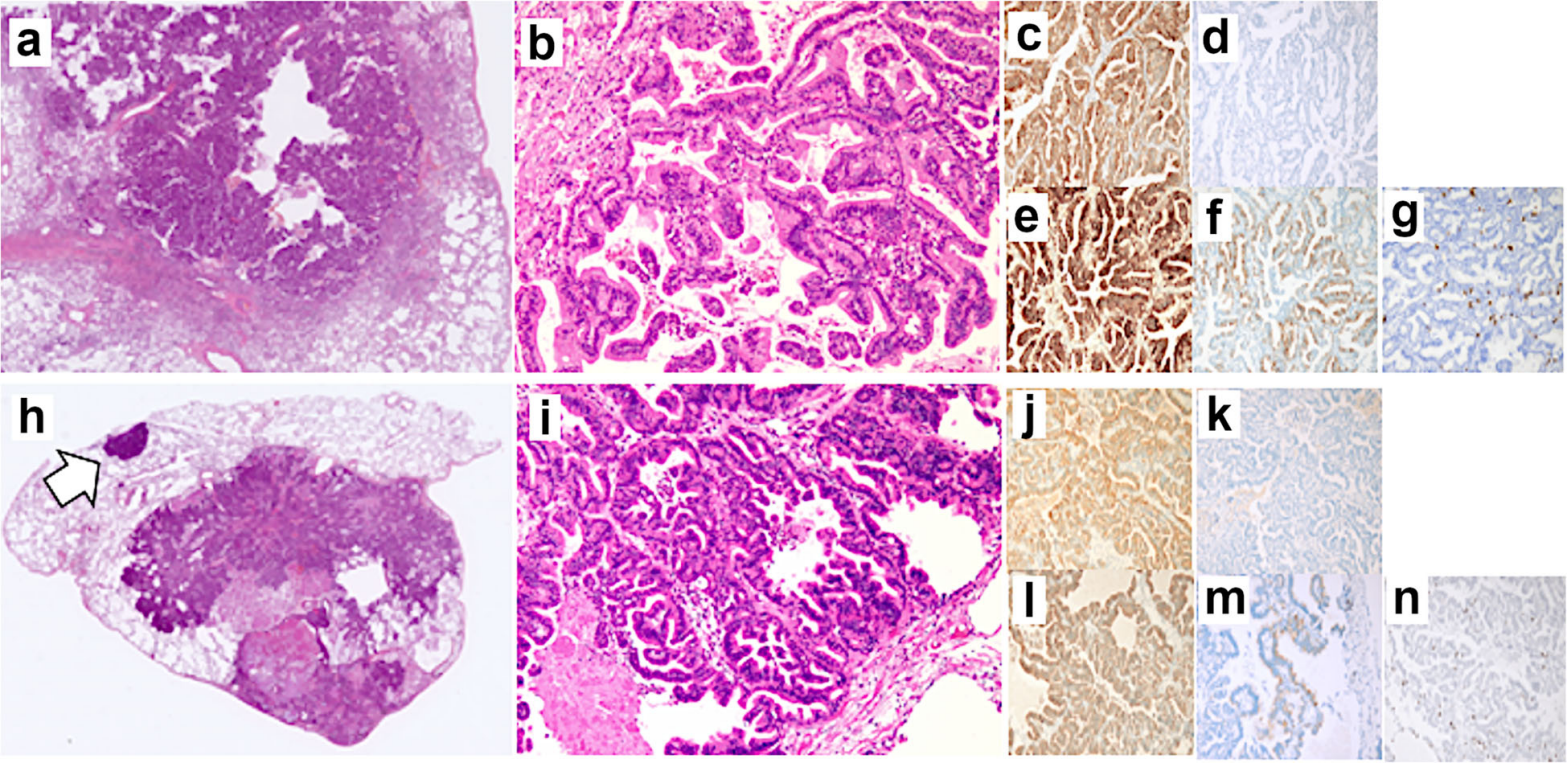
Pathological findings for both of the lung metastases. **a** Whole microscopic view showing nodular and cystic tumors in the resected right lung. **b** Tumor showing papillary, tubular, or lepidic proliferation of atypical columnar cells. Immunohistochemistry findings for the right lung specimen for **c** MUC1, **d** MUC2, **e** MUC5AC, **f** MUC6, and **g** TTF-1. **h** Whole microscopic view showing nodular and cystic tumors in the resected left lung. A small daughter lesion near the main tumor was also noted in the left lung (white arrow). **i** Tumor showing papillary, tubular, or lepidic proliferation of atypical columnar cells. Immunohistochemistry findings for the left lung specimen for **j** MUC1, **k** MUC2, **l** MUC5AC, **m** MUC6, and **n** TTF-1

Forty-two months after the first resection, a 5-mm-sized frosted glass shadow was recognized in the right lung apex (Fig. 5a). In addition, we found a 5-mm-sized node from the left superior mediastinum to the ventral pleura (Fig. 5b). Tumor markers did not increase, although we had followed up by taking chest CT scans every 4 months because we suspected inflammatory changes and/or tumor. The tumor on the right lung changed to a nodule and grew to 10 mm (Fig. 5c). We suspected lung metastasis or primary lung cancer; however, because the left lung node disappeared (Fig. 5d), we viewed it as inflammatory changes. Because of its increasing size, we strongly suspected malignant tumor (Fig. 5e, f). Serum tumor marker testing revealed elevated CEA (8.1 ng/mL) level but normal CA19-9 (33.5 U/mL) level, squamous cell carcinoma antigen (2.3 ng/mL), neuron-specific enolase (15.4 ng/mL), cytokeratin fragment (1.4 ng/mL), and pro-gastrin-releasing peptide (40.5 pg/mL). Brush cytology and biopsy on bronchoscopy revealed adenocarcinoma with negative thyroid transcription factor 1 (TTF-1) expression. From the cytohistologic features and clinical history, we presumed it to be lung metastasis from IPNB, resected in two separate procedures on the 54th month after the first resection. The size of both tumors had increased to 20 mm right before resection (Fig. 5g, h). Whole microscopic views showed nodular and cystic tumors in both tumors resected from the right and left lungs (Fig. 6a, h). A small daughter lesion near the main tumor was also noted in the left lung. Microscopically, both tumors showed papillary, tubular, or lepidic proliferation of atypical columnar cells (Fig. 6b, i). Immunohistochemistry revealed positive expression of MUC1, MUC5AC, and MUC6 but negative expression of MUC2 and TTF-1 (Fig. 6c–g, j–n) in both lung tumors. Although primary invasive mucinous carcinoma of the lung was a differential diagnosis, based on the similar histological features to previous IPNB, multiple tumor lesions in the lungs, imaging findings, and clinical history, we diagnosed it as pulmonary metastasis from IPNB. After both resections were completed, adjuvant chemotherapy was administered using tegafur (S-1) based on the recurrent lesions that we had detected. The S-1 regimen involved a dose of 120 mg/day for 4 consecutive weeks and then a 2-week break. The patient is currently alive (64 months after the first resection and 12 months after the lung resections), has completed four cycles of the S-1 regimen, and is continuing follow-up with monthly blood tests and CT evaluations every 3 months.

## Discussion and conclusion

This case involved lung metastases from IPNB that developed after a liver recurrence and 54 months after resection of the primary IPNB. The diagnosis in this case was based on hematoxylin and eosin staining and immunohistological staining. To the best of our knowledge, ours is only the third reported case of lung metastasis from IPNB, with the two other cases being described in a single-center survey by Höhn et al. [6] and in another case report [7]. TTF-1 is useful to distinguish primary lung adenocarcinoma from metastatic adenocarcinoma. TTF-1 is usually positive for primary lung adenocarcinoma but negative for adenocarcinoma of other organs [8]. However, differentiating primary invasive mucinous carcinoma of the lung from metastatic carcinoma of the bile duct or pancreas is challenging because both carcinomas shows similar histological features (papillary and lepidic growth) and frequently negative TTF-1 expression [8]. In fact, the lung tumors in this case showed papillary and lepidic growth with negative TTF-1, although we diagnosed it as IPNB metastasis based on similar histological features to previous IPNB, multiple tumor lesions in the lungs, imaging findings, and the clinical history.

The IPNB subtype (gastric, intestinal, pancreatobiliary, or oncocytic) is often considered a counterpart to intraductal papillary mucinous neoplasms in the pancreas [9–11]. Immunohistochemistry can help to determine the immunophenotype, and our findings of positive MUC1 and MUC5AC expression supported a diagnosis of the pancreatobiliary type (Fig. 2). A previous report has indicated that “MUC5AC and MUC6 are frequently expressed by the gastric-type epithelium and MUC2 by the intestinal-type. MUC1 is frequently expressed by the pancreatobiliary-type epithelium and to a variable extent by the other epithelial types” [2]. Another report has also indicated that “a significantly higher proportion of pancreatobiliary compared with intestinal tumors expressed MUC1, whereas the reverse was the case of the MUC2 antigen that was expressed in the majority of intestinal tumors but seen only in frequently in pancreatobiliary tumors” [3]. In this context, when primary and metastatic specimens exhibit the same staining pattern, the diagnoses should also be the same. A recent report from a Japanese–Korean study group categorized IPNB into type 1 (intrahepatic) and type 2 (extrahepatic) [4], and this classification method was adopted by the WHO in 2019. Nevertheless, it remains unclear how to effectively classify the subtypes of IPNB, and MUC staining patterns may not be sufficient for diagnosing a specific subtype. However, these staining patterns are likely useful for confirming that the primary and metastatic specimens are related.

In the present case, the patient underwent resection of liver recurrence at 9 months after resection of the primary tumor. Furthermore, we were able to perform resection for both lung metastases at 48 months after the liver resection. We have continued to follow the patient, and the patient is alive and without evidence of recurrence at approximately 12 months after the lung resections. This case suggests that resection may be effective for recurrence, given the patient’s long-term survival. Interestingly, the first resection had negative margins, although the patient still developed liver recurrence, which suggests that the IPNB was histologically present as a skip lesion in the preserved bile duct. The liver resection was also judged to be R0, although it is possible that the invasive component could have led to hematogenous spread, which is a possible explanation for the lung metastasis. In the CT image before the first resection, the tumor was localized in the S5. IPNB cases that produce mucus are mostly categorized as type 1, and invasive carcinoma is considered rare in comparison to type 2 [3, 12]. We doubted IPNB because mucus was grossly recognized, and images showed a papillary mass. It was difficult to determine it as benign tumor preoperatively, although we chose segmentectomy upon intraoperative diagnosis. We came across occasional reports that positron emission tomography (PET)-CT is useful in distinguishing benign and malignant in case of IPNB [12–14]. However, some argue that IPNB with small mural nodule and high amount of mucin may present as false negative [15]. In this case, we did not conduct fluorodeoxyglucose (FDG)-PET. If we had, we may have been able to determine that the first surgery should have been right hepatectomy. In addition, we had chosen a limited surgery because at the time we were not capable of operating peroral cholangioscopy or IDUS due to technical problems and because we considered IPNB a benign tumor. If we had chosen the first resection to be an extended surgery, we may have been able to prevent the hepatic relapse, and this is something we should reflect on. It is better to choose a surgery equivalent to that for a malignant tumor because it is hard to determine clear lesion localization of IPNB.

There was no clear justification for a preoperative diagnosis of lung metastasis from IPNB, which prompted us to perform bronchoscopic biopsy, and the immunostaining supported a diagnosis of IPNB metastasis and the decision to perform lung resection. The characteristics of the three reported cases with lung metastasis are summarized in Table 1. In all three cases, the initial resection was judged to be R0, and no adjuvant chemotherapy was administered. However, we treated our patient using adjuvant chemotherapy after the lung resections, based on his history of recurrence and metastasis. The two other reports did not describe the outcomes after resection of the lung metastases [6, 7], although both reports indicated that the diagnosis of metastasis was based on a comparison of the immunostaining results from the primary and lung specimens. Thus, the long-term outcomes of resection remain unclear, although our patient has achieved good survival to this point. Our patient also had a disease with an invasive component, which we suspect is associated with hematogenous spread, given the distant metastasis without lymph node involvement. In one of the previous cases, the preoperative suspicion was that the patient had lung cancer because non-invasive IPNB without lymph node metastasis is extremely rare; however, after the genomic analysis of the resected specimen, IPNB metastasis was confirmed. Thus, the authors suggested that the mechanism underlying metastasis is actually more complex than what was previously thought [7]. Performance status and age may be relevant when considering the resection of IPNB recurrence and/or metastasis; using appropriate surgical technique and careful case selection will not only allow better determination of long-term outcomes but also help to clarify the mechanism underlying metastasis by analysis of surgical specimens.

**Table 1:** Reported cases of lung metastases from intraductal papillary neoplasm of the bile duct

Case	Year	Author	First resected specimen	IPNB type	N	R	V	Immunosubtype	MUC 1 expression	Adjuvant therapy after first resection
1	2019	Höhn et al. [6]	pT2N0M0 invasive	1	0	0	0	Pancreatobiliary	Unknown	None
2	2019	Nam et al. [7]	pTisN0M0 non-invasive	2	0	0	0	Intestinal	Unknown	None
3	2020	Present case	pT2N0M0 invasive	1	0	0	1	Pancreatobiliary	Positive	None
Case	Preoperative diagnosis of lung lesion			Diagnostic modality for lung metastasis				Time to lung metastasis	Adjuvant therapy after resection of lung metastasis	
1	Metastasis from IPNB			Biopsy and immunohistochemistry				9 months	FOLFOX	
2	Lung cancer (T1bN0M1b)			Genomic profiling analysis and whole exome sequencing				32 months	S-1	
3	Metastasis from IPNB			Biopsy and immunohistochemistry				54 months	S-1	

*IPNB* intraductal papillary neoplasm of the bile duct, *FOLFOX* oxaliplatin, folic acid, and 5-fluoruracil, *S-1* tegafur

We are only aware of a few case reports regarding metastatic IPNB because it has a relatively benign course [7, 16]. Most of these reports have involved small retrospective studies, although Gordon-Weeks et al. reported a systematic review and meta-analysis in 2016 [2]. The results indicated that recurrence occurred in 13–29% of cases after curative resection, and this proportion increased to 47–62% if the patient had invasive disease [17–19]. While the classification of IPNB remains complex, meta-analyses and multi-center cohort studies are gradually clarifying the outcomes and prognoses [2, 5, 6, 20, 21], and there is also increasing information regarding the surveillance protocols for IPNB and appropriate postoperative follow-up [5, 6, 22]. The present case involved an initial pathological diagnosis of invasive carcinoma with a MUC1-expressing pancreatobiliary type, which is a known prognostic factor. Based on the 2019 WHO classification, cholangiocarcinoma derived from IPNB has a 5-year recurrence rate of 47.0% and a 5-year overall survival rate of 68.8% [3]. In addition, the pancreatobiliary type has poorer clinical outcomes than the other subtypes, while MUC1 expression is associated with shorter recurrence-free survival [3]. Although IPNB is a slow-growing tumor, it has significant potential for progression to invasive disease, and Höhn et al. strongly suggested a short-interval follow-up even if benign IPNB has been completely resected [6]. In case of invasive IPNB, there is a good chance of expecting long-term survival by resection even if any lesion of recurrence/metastasis occurs. Therefore, we need to conduct a follow-up at short-term intervals, as suggested by Höhn et al. However, there is no clear difference in the treatment strategy for IPNB recurrence or metastasis, and further information is needed to determine whether these lesions need to be treated differently.

CT images showed different types of lung lesions (left and right). The lesion on the left lung decreased once its size, but showed growth afterwards, which confused our judgment. We followed up for nearly a year based on the acknowledgement that IPNB is a benign tumor but decided on resection upon recognition of the growth of the lung tumor. Generally, the lung is a minor site of recurrence in cholangiocarcinoma (CC), and chemotherapy is the first choice in case of intrahepatic CC lung metastasis. In CC, the lung metastasis rate after resection is reported to range from 1.6 to 11.7% [23–25]. In contrast, there are only a few reports in relation to IPNB metastasis. As for lung metastasis, reports by You et al. [5] reported one case (1.3%) of lung metastasis out of 74 cases of relapsed patients. IPNB has a better prognosis than conventional bile duct CC [26, 27]. Although IPNB has low malignancy (especially type 1), the rate of relapse in infiltrating cancer is around 47–62% [2, 3]. In case of a relapse or metastasis, systemic chemotherapy according to the biliary tract cancer is appropriate. Approved medication in Japan for the said cancer would be gemcitabine, tegafur, and cisplatin, of which a combination has not been established as an effective regimen in cases of adjuvant therapy for unresectable/relapsed biliary tract cancer. Beginning with BILCAP trials [28], there are several reports [29, 30] on the efficaciousness of chemotherapy for biliary tract cancer and recent reports on the efficaciousness of nab-paclitaxel [31]. Chemotherapy needs to be chosen properly, considering the performance status, age, and/or medical history of the patient. We expect further chemotherapy regimens for biliary tract cancer in the future. Because the disease concept of IPNB is relatively new, it is highly possible that something previously diagnosed as intrahepatic CC is now diagnosed as IPNB. We expect further collection of IPNB reports in the future.

In conclusion, we encountered a rare case of lung metastases from IPNB, which were diagnosed immunohistologically. Given that IPNB is a slow-growing tumor, recurrence and/or metastasis may be detected during long-term follow-up. In case of invasive IPNB, there is a good chance of long-term survival by resection even if recurrence/metastasis occurs by conducting a follow-up at short-term intervals. It is also necessary to continue collecting information regarding the most effective techniques for managing these patients.

## Data Availability

All data generated or analyzed during this study are included in this published article.

## Abbreviations

CA19-9: Cancer antigen 19-9
CC: Cholangiocarcinoma
CEA: Carcinoembryonic antigen
CT: Computed tomography
ERC: Endoscopic retrograde cholangiography
IDUS: Intraductal ultrasonography
IPNB: Intraductal papillary neoplasm of the bile duct
S5: Segment 5
TTF-1: Thyroid transcription factor 1
WHO: World Health Organization
